# An acute temperature rise to 40°C inhibits free fatty acid uptake into white adipocytes

**DOI:** 10.1080/21623945.2026.2626121

**Published:** 2026-02-11

**Authors:** Federica Foti, Raoul S. Schaepper, Daniel Konrad, Stephan Wueest

**Affiliations:** a Division of Pediatric Endocrinology and Diabetology, University Children’s Hospital, University of Zurich, Zurich, Switzerland; b Children’s Research Center, University Children’s Hospital, University of Zurich, Zurich, Switzerland; c Zurich Center for Integrative Human Physiology, University of Zurich, Zurich, Switzerland

**Keywords:** Fever, free fatty acid uptake, lipolysis, temperature, white adipose tissue

## Abstract

Fever reflects a physiological rise in body temperature accompanied by elevated production of adrenaline. The increased body temperature in fever is caused by shivering thermogenesis in skeletal muscle and non-shivering thermogenesis in brown adipose tissue (BAT), the latter being mediated by uncoupled oxidation of free fatty acids (FFAs). We hypothesized that an acute temperature rise to 40°C increases adrenalin-induced lipolysis in white adipocytes, thereby potentially providing FFAs as an energy substrate to sustain fever-induced thermogenesis in skeletal muscle and BAT. In 3T3-L1 and primary murine white adipocytes, isoproterenol-induced extracellular FFA accumulation was significantly increased at 40°C compared to 37°C. In contrast, isoproterenol-induced increase in extracellular glycerol concentrations and the protein levels of phosphorylated hormone sensitive lipase were comparable at both temperatures, suggesting a similar degree of lipolysis. Moreover, incubation at 40°C did neither increase isoproterenol-induced oxygen consumption nor intracellular FFA concentrations, indicating that the elevated extracellular FFA accumulation was not due to reduced intracellular consumption. Conversely, isoproterenol blunted FFA uptake into adipocytes to a significantly higher extent at 40°C compared to 37°C. Hence, an acute temperature rise to 40°C reduces FFA uptake into white adipocytes, thereby increasing extracellular FFA availability.

## Introduction

Fever reflects a physiological stress-response characterized by increased body temperature that is paralleled by elevated production of the beta-adrenergic receptor agonist adrenaline [1,2]. The increased body temperature in fever is caused by shivering thermogenesis in skeletal muscle and non-shivering thermogenesis in brown adipose tissue (BAT), the latter being mediated by uncoupled oxidation of free fatty acids (FFAs) [3]. FFA supply for thermogenesis may be provided by white adipose tissue (WAT), a central player in lipid metabolism, storing energy in the form of triglycerides (TG). Adrenalin-induced beta-adrenergic signalling is an important mediator of FFA mobilization through activation of lipolysis. Adipose triglyceride lipase (ATGL) and hormone sensitive lipase (HSL) are key enzymes in this process contributing to the hydrolytic cleavage of fatty acids from TG, resulting in the release of FFAs and glycerol [4,5]. Following hydrolytic cleavage, FFAs may be used for energy consumption (i.e. beta oxidation) within adipocytes. Alternatively, FFAs may be co-secreted with glycerol into the extracellular compartment, where they serve as energy substrates for other cells and organs. Alternatively, secreted FFAs can re-enter adipocytes for internal storage or energy production [4–6].

Herein, we hypothesized that fever (i.e. an acute temperature rise to 40°C) increases adrenaline-induced lipolysis in white adipocytes, thereby providing FFAs as an energy substrate for fever-induced thermogenesis. Of note, fever is not only paralleled by increased adrenaline but also by elevated inflammatory cytokine production [1,2]. To avoid the confounding endocrine effects of inflammatory cytokines on lipolysis [7], experiments were performed in cultured white adipocytes and in primary white adipocytes *ex vivo* (i.e. in the absence of immune cells).

## Results

### Increased isoproterenol-induced extracellular FFA accumulation in 3T3-L1 adipocytes incubated at 40°C

**Figure 1.**
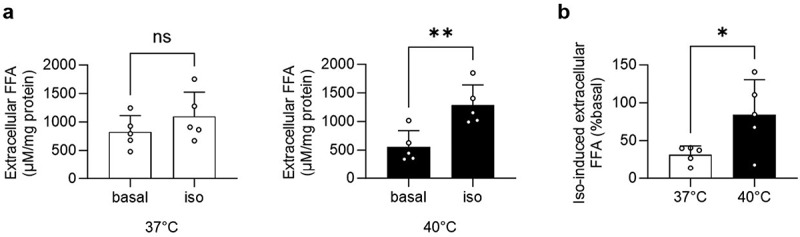
Increased isoproterenol-induced extracellular FFA accumulation in 3T3-L1 adipocytes incubated at 40°C.

To address whether an acute temperature rise increases beta-adrenergic signalling-induced FFA release, mature 3T3-L1 adipocytes were incubated for 1 h at 37°C or 40°C with or without the beta-adrenergic receptor agonist isoproterenol. While isoproterenol did not change extracellular FFA concentration significantly at 37°C, it significantly increased extracellular FFA accumulation at 40°C (Figure 1(a)). Consequently, isoproterenol-induced increase in extracellular FFA accumulation was significantly higher at 40°C (Figure 1(b)). In contrast, the inhibitory effect of insulin on FFA release did not differ between 3T3-L1 adipocytes incubated at 37°C and 40°C (Supplementary Figure. S1(a)). Of note, no temperature-mediated difference in isoproterenol-induced extracellular FFA accumulation was observed in a mouse-derived subcutaneous white adipocyte cell line [8] (Supplementary Figures. S1(b and c)).

Extracellular FFA concentration in mature 3T3-L1 adipocytes treated with vehicle control (basal) or 1 µM isoproterenol (iso) and incubated at 37°C or 40°C for 1 h. Shown are absolute values normalized to protein (a) and iso-induced FFA concentrations (% basal) (b). *n* = 5 independent cell culture experiments. *p < 0.05, **p < 0.01. Statistical test used: unpaired Student’s *t* test (a and b). Data are shown as mean±SEM.

### Increased isoproterenol-induced extracellular FFA accumulation in primary white adipocytes incubated at 40°C

**Figure 2.**
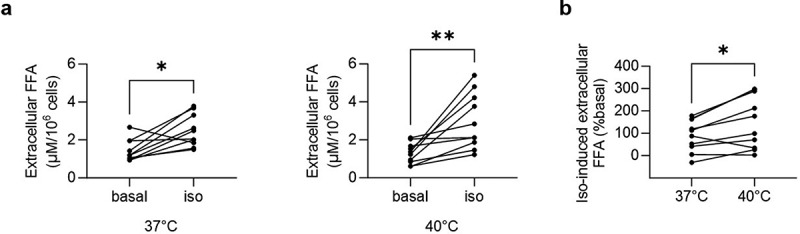
Increased isoproterenol-induced extracellular FFA accumulation in primary white adipocytes incubated at 40°C.

Next, we aimed to assess the effect of an acute temperature rise on FFA accumulation in primary white adipocytes. Since the latter was not affected in cultured subcutaneous adipocytes (Supplementary Figures. S1(b and c)), this experiment was performed using intra-abdominal adipocytes, a depot that is more responsive towards adrenalin-induced lipolysis compared to subcutaneous fat cells [9]. To this end, perigonadal adipocytes were isolated from chow-fed C57BL/6J mice and incubated at 37°C or 40°C for 1 h in the presence or absence of isoproterenol. While isoproterenol significantly increased extracellular FFA accumulation at both temperatures (Figure 2(a)), its effect was significantly higher at 40°C (Figure 2(b)). Conversely, the inhibitory effect of insulin was similar at both temperatures (Supplementary Figure. S2). Hence, an acute temperature rise from 37°C to 40°C increased beta-adrenergic signalling-induced extracellular FFA accumulation in cultured 3T3-L1 as well as primary white adipocytes, without affecting insulin-inhibited FFA release.

Extracellular FFA concentration in primary white adipocytes treated with vehicle control (basal) or 1 µM isoproterenol (iso) and incubated at 37°C or 40°C for 1 h. Shown are absolute values normalized to cell number (a) and iso-induced FFA concentrations (% basal) (b). *n* = 10 mice. *p < 0.05, **p < 0.01. Statistical test used: Wilcoxon test (a; left panel), paired Student’s *t* test (a, right panel; b).

### Similar isoproterenol-induced extracellular glycerol accumulation in white adipocytes incubated at 37°C and 40°C

**Figure 3.**
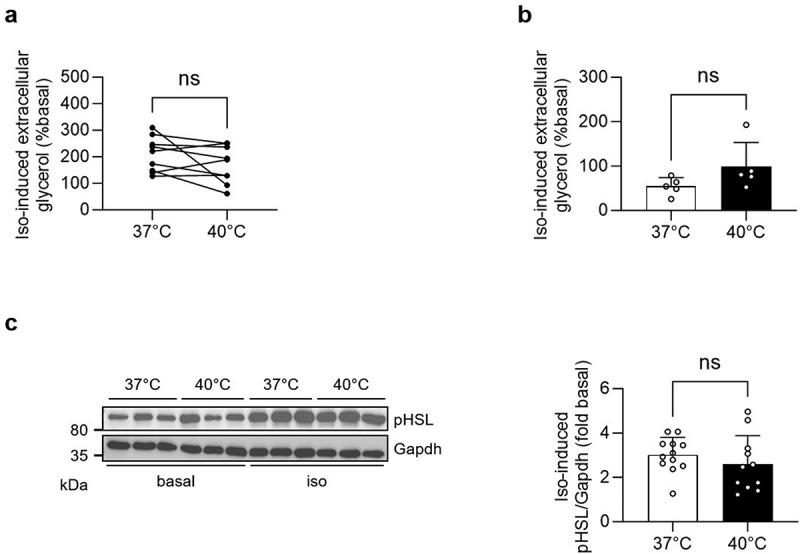
Similar isoproterenol-induced extracellular glycerol accumulation in white adipocytes incubated at 37°C and 40°C.

Since we hypothesized that an acute temperature rise increases beta-adrenergic signalling-induced lipolysis, extracellular glycerol levels were analysed next. While isoproterenol significantly increased extracellular glycerol concentrations in both primary (Supplementary Figure. S3(a)) and 3T3-L1 adipocytes (Supplementary Figure. S3(b)) at both temperatures, the effect of isoproterenol was not increased at 40°C neither in primary (Figure 3(a)) nor in 3T3-L1 (Figure 3(b)) adipocytes. Isoproterenol significantly elevated phosphorylation of HSL in 3T3-L1 adipocytes at both temperatures, however to a similar degree (Figure 3(c) and Supplementary Figure. 3(c)). These results suggest that increased beta-adrenergic signalling-induced extracellular FFA accumulation at 40°C may not be mediated by elevated lipolysis. Furthermore, the fact that lipolysis was similar at both temperatures indicates that viability was maintained at 40°C. Likewise, the unchanged protein amount (129.3 ± 12.5 µg/well at 37°C *vs*. 135.8 ± 12.9 µg/well at 40°C, *p* = 0.68) suggests that the acute rise in temperature did not increase apoptosis.

Extracellular glycerol concentration in primary white adipocytes (a) and mature 3T3-L1 adipocytes (b) treated with vehicle control (basal) or 1 µM isoproterenol (iso) and incubated at 37°C or 40°C for 1 h. Shown is iso-induced glycerol concentration (% basal). *n* = 9 mice (a) and *n* = 5 independent cell culture experiments (b). (c) Representative Western blot and quantification of iso-induced phospho(p)HSL protein levels of 3T3-L1 adipocytes treated with vehicle control (basal) or 1 µM isoproterenol (iso) and incubated at 37°C or 40°C for 1 h. *n* = 11–12 wells of 4 independent cell culture experiments (2–3 wells per experiment). Data are shown as mean±SEM (for b and c). Statistical test used: paired Student’s *t* test (a), unpaired Student’s *t* test (b and c).

### Decreased FFA uptake in white adipocytes incubated at 40°C

**Figure 4.**
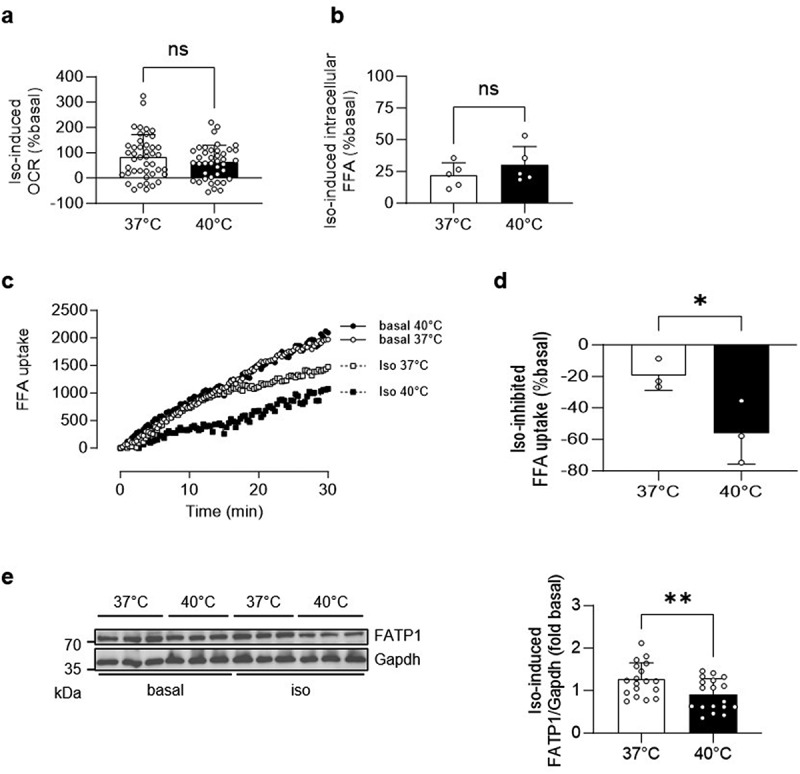
Decreased FFA uptake in white adipocytes incubated at 40°C.

To investigate whether decreased intracellular consumption (i.e. lower beta-oxidation) contributes to elevated extracellular FFA accumulation at 40°C, experiments using a Seahorse analyser were performed. Isoproterenol increased oxygen consumption rate (OCR) in 3T3-L1 adipocytes at both temperatures, resulting in a comparable isoproterenol-induced increase in OCR (Figure 4(a) and Supplementary Figure. S4(a)). Hence, augmented extracellular FFA accumulation at 40°C may not result from reduced intracellular free fatty acid consumption. Consistently, the isoproterenol-induced increase in intracellular FFA concentration was not significantly higher at 40°C (Figure 4(b) and Supplementary Figure. S4(b)). Based on these data, we speculate that reduced free fatty acid uptake at 40°C may cause increased extracellular FFA accumulation. Therefore, we measured the uptake of BODIPY labelled fatty acids into 3T3-L1 adipocytes that were incubated at either 37°C or 40°C, with or without isoproterenol. Compared to control cells, isoproterenol decreased FFA uptake at both temperatures (Figure 4(c)). However, the isoproterenol-mediated decrease in FFA uptake was significantly elevated at 40°C compared to 37°C (Figure 4(d)), indicating that blunted FFA uptake into adipocytes leads to increased isoproterenol-induced extracellular FFA accumulation at 40°C. Since fatty acid transport protein 1 (FATP1) is temperature-sensitive [10], we hypothesized that reduced concentrations of this FFA transporter contribute to blunted FFA uptake at 40°C. Indeed, an acute temperature rise lowered the isoproterenol-induced increase in FATP1 protein levels (Figure 4(e) and Supplementary Figure. S4(c)).

(**a**) Oxygen consumption rate (OCR) in mature 3T3-L1 adipocytes treated with vehicle control (basal) or 1 µM isoproterenol (iso) and incubated at 37°C or 40°C for 1 h. Shown is iso-induced increase in OCR (% basal). *n* = 40–44 wells of 2 independent cell culture experiments (17–23 wells per experiment). (**b**) Intracellular FFA concentration in mature 3T3-L1 adipocytes treated with vehicle control (basal) or 1 µM isoproterenol (iso) and incubated at 37°C or 40°C for 1 h. Shown is iso-induced FFA concentration (% basal). *n* = 5 independent cell culture experiments. FFA uptake in mature 3T3-L1 adipocytes was measured in cells after being treated with vehicle control (basal) or 1 µM isoproterenol (iso) and incubated at 37°C or 40°C for 1 h. Shown is (**c**) FFA uptake over 30 minutes and (**d**) % iso-inhibited FFA uptake (calculated as % basal of the area under the curve (AUC)). *n* = 3 independent cell culture experiments. (**e**) Representative Western blot and quantification of iso-induced FATP1 protein levels of 3T3-L1 adipocytes treated with vehicle control (basal) or 1 µM isoproterenol (iso) and incubated at 37°C or 40°C for 1 h. *n* = 18 wells of 6 independent experiments (3 wells per experiment). *p < 0.05, **p < 0.01. Statistical test used: unpaired Student’s *t* test (a, b, d and e). Data are shown as mean (c) or mean±SEM (a, b, d and e).

## Discussion

Herein, we hypothesized that an acute temperature rise to 40°C would increase adrenaline-induced lipolysis in WAT, thereby providing FFAs as energy substrate to sustain fever-induced thermogenesis. In line with our hypothesis, elevating the temperature from 37°C to 40°C increased isoproterenol-induced extracellular FFA accumulation in 3T3-L1 and primary murine white adipocytes. However, insulin-inhibited FFA release remained unchanged, suggesting that an acute temperature rise affects stimulatory but not inhibitory pathways that regulate extracellular FFA accumulation. Elevated temperature had no effect on isoproterenol-induced extracellular FFA accumulation in white subcutaneous adipocytes. This may be explained by reduced metabolic activity of subcutaneous adipocytes. Indeed, catecholamine-induced lipolysis may be blunted in subcutaneous compared to intra-abdominal adipocytes [9].

Contrary to our hypothesis, an acute temperature rise may not affect beta-adrenergic signalling-induced lipolysis. While isoproterenol significantly increased lipolysis (i.e. pHSL protein levels and extracellular glycerol concentrations) at both 37°C and 40°C, the stimulatory effect of isoproterenol was similar between both temperatures. Nevertheless, we cannot rule out that subtle changes in ATGL/HSL activity as well as differences in FFA re-esterification and/or cycling may have contributed to the observed effect. Previous studies suggested that β-adrenergic receptor kinetics and agonist affinity are temperature dependent [11,12]. While we did not observe an effect of an acute temperature increase on beta-adrenergic signalling involved in lipolysis, other cellular responses regulated by beta-adrenergic singling may still be changed at 40°C.

An acute temperature rise did not affect isoproterenol-induced oxygen consumption or intracellular FFA concentrations. This indicates that increased extracellular accumulation of free fatty acids at 40°C does not result from reduced intracellular consumption. Rather, the observed divergence between intra- and extracellular concentrations suggests that FFAs increasingly accumulate outside of cells at 40°C due to reduced FFA uptake. Indeed, we provide evidence that reduced FFA uptake plays a key role in this process. Of note, isoproterenol reduced free fatty acid uptake into white adipocytes at both 37°C and 40°C. This suggest that activation of beta-adrenergic signalling increases FFA mobilization from white adipocytes not only via inducing lipolysis, but also via reducing FFA re-uptake. Importantly, such inhibiting effect of isoproterenol on FFA uptake was enhanced at 40°C, leading to higher extracellular FFA concentrations. Consequently, increased FFA mobilization potentially sustains fever-induced thermogenesis in BAT and/or skeletal muscle. The uptake of FFAs into adipocytes is mediated by proteins such as FATP1. FATP1 has been shown to be temperature-sensitive, with cold temperatures increasing its content in BAT and thereby augmenting FFA uptake [10]. Similarly, we found that an acute temperature increase reduces the isoproterenol-induced increase in FATP1 in white adipocytes. This may have contributed to the inhibition of FFA uptake caused by a temperature increase to 40°C. Clearly, further research is needed to establish whether abundance, activity or localization of FFA transporters is critically involved in the accumulation of FFAs at 40°C. Additionally, temperature-dependent changes in membrane fluidity or endocytosis may have also contributed to the observed changes.

A temperature of 40°C reflects high-grade fever [13], representing a rather extreme and acute condition. Whether moderate fever (38.5–39°C) has similar effects on FFA metabolism remains to be investigated. Since the temperature of intra-abdominal WAT may be similar to core body temperature [14], adipocytes therein are presumably exposed to rising temperatures during fever. However, to the best of our knowledge, WAT temperature has never been assessed *in vivo*, so it needs to be confirmed that it reaches 40°C in high-grade fever. Furthermore, it is necessary to investigate whether an acute temperature rise increases extracellular FFA accumulation *in vivo*, and, if so, whether it plays a critical role in whole-body metabolism.

In conclusion, an acute temperature rise blunts free fatty acid uptake into white adipocytes, thereby increasing extracellular FFA accumulation. Such temperature-mediated lipid mobilization from white adipocytes may help to cover elevated energy demand during fever.

## Methods

### FFA and glycerol release from white adipocytes

3T3-L1 and subcutaneous white adipocytes were seeded and differentiated as recently described [15]. Mature cultured white adipocytes were incubated for 1 h in Krebs Ringer phosphate-HEPES buffer (KRB) supplemented with 0.1% fatty acid-free BSA in a non-CO_2_ incubator (without monitoring pH changes) either at 37°C or 40°C in the presence or absence of 1 µM isoproterenol Sigma-Aldrich (Merck Group, St. Louis, MO, USA) or 100 nmol/l insulin (Actrapid®, Novo Nordisk, Bagsværd, Denmark). Primary white adipocytes were isolated from 18-week-old male mice on a C57BL/6J background as previously reported [16]. Samples size was decided based on previous experience in the laboratory. Experiments were performed in 10 mice on 3 days. Animal experiments were conformed to the Swiss animal protection laws and were approved by the Cantonal Veterinary Office in Zurich, Switzerland (Licence ZH088/2024). ARRIVE reporting guidelines were used. Mice had ad libitum access to standard chow diet (Kliba Nafag, Kaiseraugst, Switzerland, #3436, 4.5% crude fat) and water and were kept in a 12 h: 12 h light: dark cycle (light phase starting at 7.00am) with ambient temperature kept at 22°C in a pathogen-free animal facility. Individually ventilated cages were enriched with crinklets and gentle tunnel handling was used. Animals were monitored weekly (habitus, fur, activity, locomotion, eye symptoms, body weight). Mice were euthanized by CO_2_ asphyxiation and the abdominal cavity opened to expose perigonadal adipose tissue. After isolation, adipocytes were incubated for 1 h in KRB supplemented with 1 mM D-Glucose and 1% fatty acid-free BSA in a water bath at 37°C or 40°C in the presence or absence of 1 µM isoproterenol Sigma-Aldrich or 100 nmol/l insulin. FFA and glycerol release was measured with (isoproterenol, insulin) or without (basal) interventions in adipocytes isolated from the same mouse. There was no randomization used to allocate primary adipocytes to intervention groups and order of treatment was the same for each mouse. Experimenters were not blinded to allocations of interventions and each mouse was considered an experimental unit. FFA and glycerol concentration in KRB was determined using a WAKO kit (Fujifilm, Tokyo, Japan) and a kit (F6428) from Sigma-Aldrich, respectively. FFA and glycerol release was normalized to the number of incubated adipocytes (assessed using a haemocytometer).

### Mitochondrial respiration

3T3-L1 cells were seeded (7,000/well) onto gelatin-coated 96-well Seahorse cell culture microplates with moats and differentiated as described above. Subsequently, medium was replaced by Seahorse XF DMEM Medium pH 7.4 supplemented with 5.5 mM glucose, 4 mM glutamine, 1 mM pyruvate and 2% FFA-free BSA. The plate was degassed in a non-CO_2_ incubator at 37°C or 40°C for 1 h. Oxygen consumption rate (OCR) was measured in a Seahorse XF Pro Extracellular Flux Analyzer (Agilent Technologies, Santa Clara, CA, USA) at 37°C or 40°C, before and after treatment with oligomycin (5 µM) and antimycin A (5 µM). Basal oxygen consumption rate was calculated as last measurement before oligomycin injection minus minimum measurement after antimycin A injection.

### FFA uptake

FFA uptake was performed using the QBT^tm^ Fatty Acid Uptake Kit from Molecular Devices (San Jose, CA, USA) according to manufacturer’s description. In brief, 50,000 mature 3T3-L1 adipocytes per well were seeded into a gelatin coated 96 well plate (black with clear bottom) and centrifuged at 1,000 rpm for 3 min with brake off. Control wells (blank) contained no cells but were otherwise treated like wells containing adipocytes. Plates were incubated for 5 h at 37°C in a CO_2_ incubator, with serum depletion for the last hour. Subsequently, media was replaced by KRB supplemented with 0.1% fatty acid-free BSA and plates were incubated for 1 h in a non-CO_2_ incubator at 37°C or 40°C in the presence or absence of 1 µM isoproterenol (4–6 wells per condition). Thereafter, KRB was removed, loading buffer was added and plate was immediately read for 30 min with readings every 20 s (excitation filter 484 nm, emission filter 515 nm) on a multimode reader (Cytation 5, Agilent). Average of blank wells was subtracted from each sample well (4–6 replicates per experiment) per time point, and emission at time point 0 was subtracted from each subsequent time point for each sample well. Area under the curve (AUC) was calculated to assess the inhibitory effect of isoproterenol on FFA uptake.

### Protein isolation and western blotting

Proteins were extracted in RIPA buffer supplemented with phosphatase and proteinase inhibitors. Protein concentration was determined with the Pierce BCA Protein Assay Kit (ThermoFisher Scientific). Details on Western blotting procedures were previously described [17]. In brief, equal quantities of protein were resolved using SDS-PAGE and transferred onto nitrocellulose membranes, blocked in 5% dry fat milk, incubated with primary antibodies diluted in 0.1% TBST at 4°C overnight and subsequently with corresponding secondary antibodies for 1 h at room temperature. Developed membranes were imaged with a ChemiDoc MP Imaging System (BioRad, Hercules, CA, USA) and quantified with ImageLab software (BioRad, version 5.2.1). The following primary antibodies were used (diluted 1:1,000): pHSL #4139 (Cell Signalling); FATP1 sc-25541 (Santa Cruz Biotechnology); Gapdh, G9545 (Sigma-Aldrich).

### Data analysis

Data are presented as means ± SEM and were analysed by unpaired (cell culture experiments) or paired (primary adipocytes) two-tailed Student’s *t* test (for normally distributed data), Mann–Whitney (cell culture experiments) or Wilcoxon matched-pairs signed rank (primary adipocytes) test (for not normally distributed data) or two-way ANOVA with Tukey’s multiple comparisons. Outliers defined by the ROUT test were excluded from statistical analysis, a criteria that was established a priori. Statistical tests were calculated using GraphPad Prism 8.00 (GraphPad Software, San Diego, CA, USA). *p* values < 0.05 were considered to be statistically significant.

## Supplementary Material

Supplementary Figures Foti et al_revised_final_not highlighted.pdf

## Data Availability

All data supporting the findings of this study are available within the article, its Supplementary Figures or under https://doi.org/10.5281/zenodo.18270174.
