# Supplementary material for: An acute temperature rise to 40°C inhibits free fatty acid uptake into white adipocytes
Source: Adipocyte. 2026 Feb 11;15(1):2626121. doi: 10.1080/21623945.2026.2626121 (PMC12915836; doi:10.1080/21623945.2026.2626121)
Supplement: Supplementary Figures Foti et al_revised_final_not highlighted.pdf [file KADI_A_2626121_SM3142.pdf]

An acute temperature rise to 40°C inhibits free fatty acid uptake into white adipocytes

Federica Foti, Raoul S. Schaepper, Daniel Konrad, Stephan Wueest

Appendix

Supplementary Figures 1-4

Supplementary Figure 1

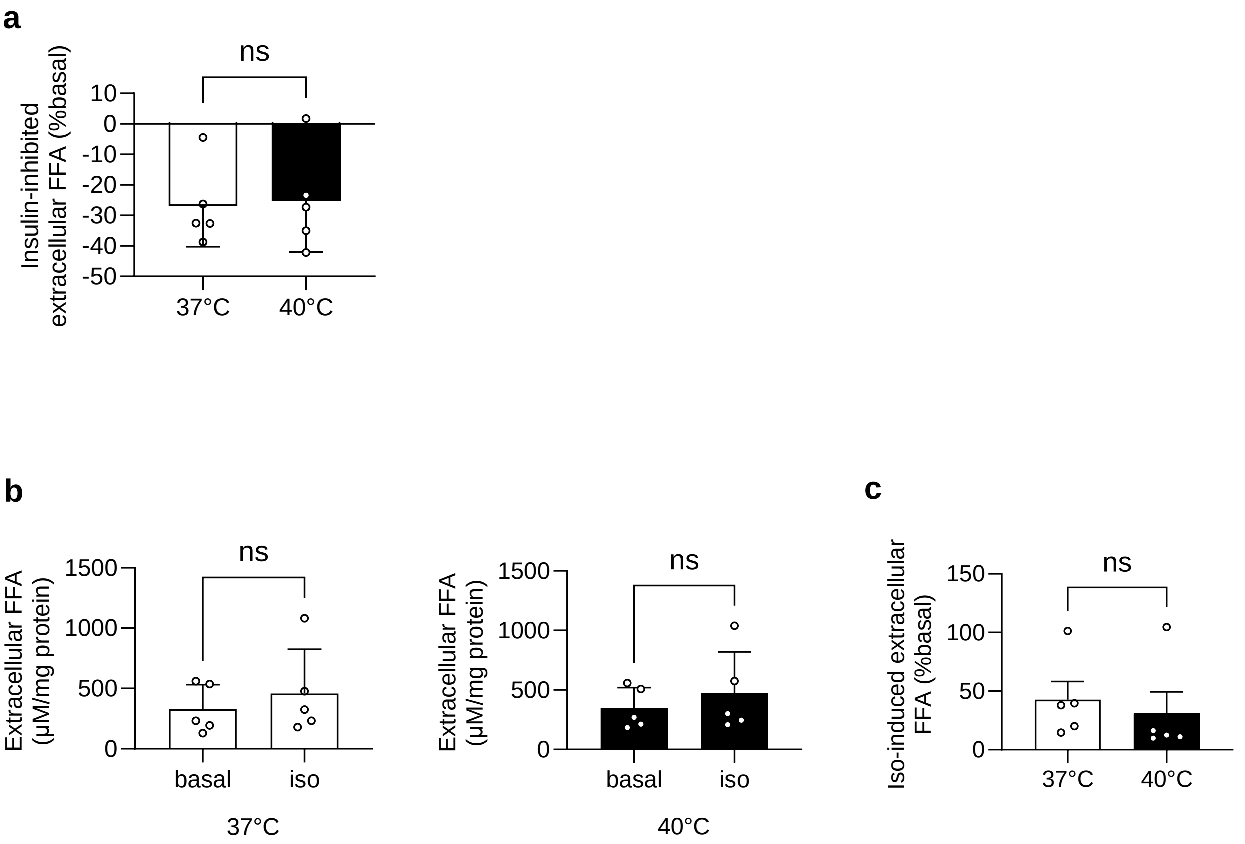

Similar insulin-inhibited extracellular FFA accumulation in 3T3-L1 adipocytes incubated at 40°C
(**a**) Extracellular FFA concentration in mature 3T3-L1 adipocytes treated with vehicle control (basal) or 100 nmol/l insulin and incubated at 37° or 40°C for 1 hour. Shown is insulin-inhibited FFA concentration (% basal). n=5 independent experiments. Extracellular FFA concentration in subcutaneous white adipocytes treated with vehicle control (basal) or 1 µM isoproterenol (iso) and incubated at 37° or 40°C for 1 hour. Shown are absolute values normalized to protein (**b**) and iso-induced FFA concentrations (% basal) (**c**). n=5 independent cell culture experiments. Statistical test used: unpaired Student's *t* test (a and b), Mann-Whitney (c). Data are shown as mean±SEM.

Supplementary Figure 2

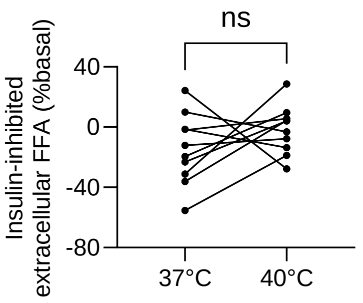

Similar insulin-inhibited extracellular FFA accumulation in primary white adipocytes incubated at 40°C

Extracellular FFA concentration in primary white adipocytes treated with vehicle control (basal) or 100 nmol/l insulin and incubated at 37° or 40°C for 1 hour. Shown is insulin-inhibited FFA concentration (% basal). n=10 mice. Statistical test used: paired Student's *t* test.

Supplementary Figure 3

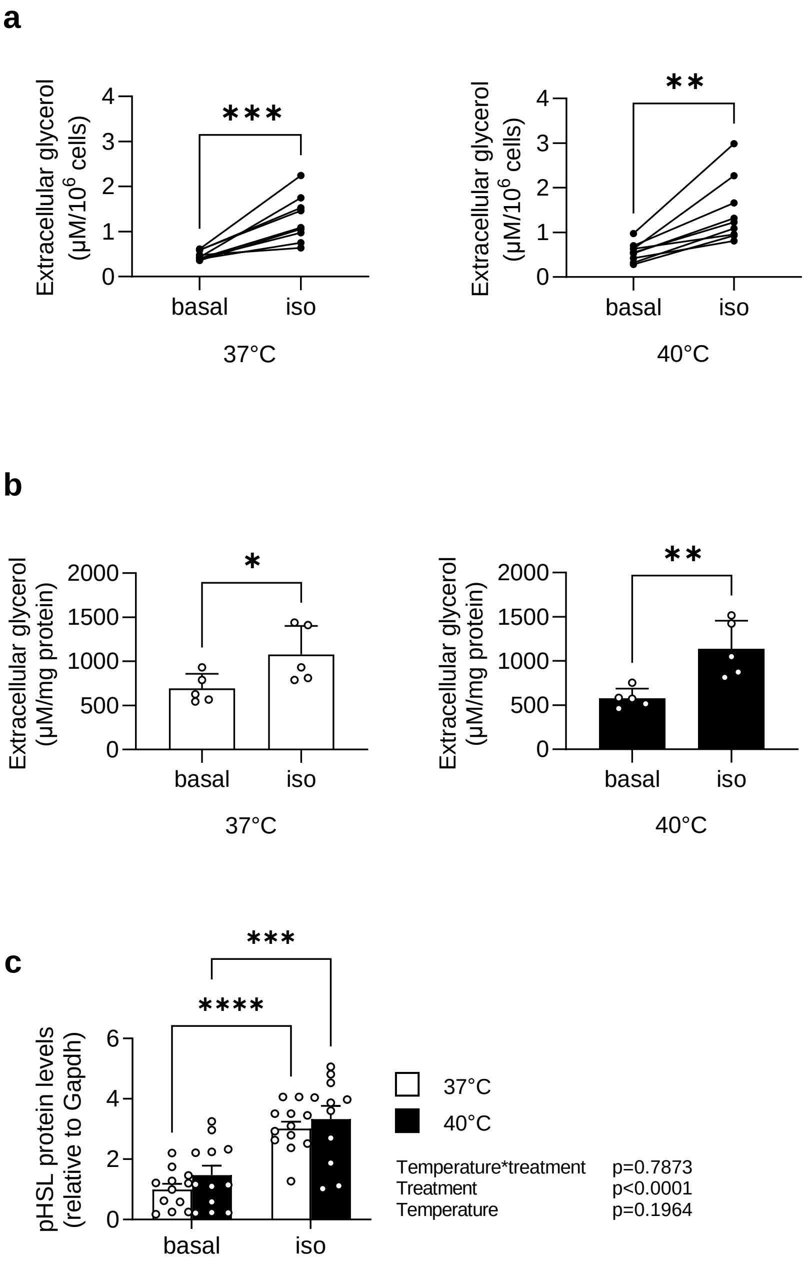

Similar extracellular glycerol accumulation in adipocytes incubated at 40°C

(**a**) Extracellular glycerol concentration in primary white adipocytes treated with vehicle control (basal) or 1 µM isoproterenol (iso) and incubated at 37° or 40°C for 1 hour. Shown are absolute values normalized to cell number. n=9 mice. **p<0.01, ***p<0.001. (**b**) Extracellular glycerol concentration in mature 3T3-L1 adipocytes treated with vehicle control (basal) or 1 µM isoproterenol (iso) and incubated at 37° or 40°C for 1 hour. Shown are absolute values normalized to protein. n=5 independent cell culture experiments. *p<0.05, **p<0.01. (**c**) Quantification of pHSL protein levels of 3T3-L1 adipocytes treated with vehicle control (basal) or 1 µM isoproterenol (iso) and incubated at 37° or 40°C for 1 hour. n=11-12 wells of 4 independent cell culture experiments (2-3 wells per experiment). ***p<0.001, ****p<0.0001. Statistical test used: paired Student’s *t* test (a), unpaired Student’s *t* test (b), two-way ANOVA with Tukey’s multiple comparisons (c). Data are shown as mean±SEM (for b and c).

Supplementary Figure 4

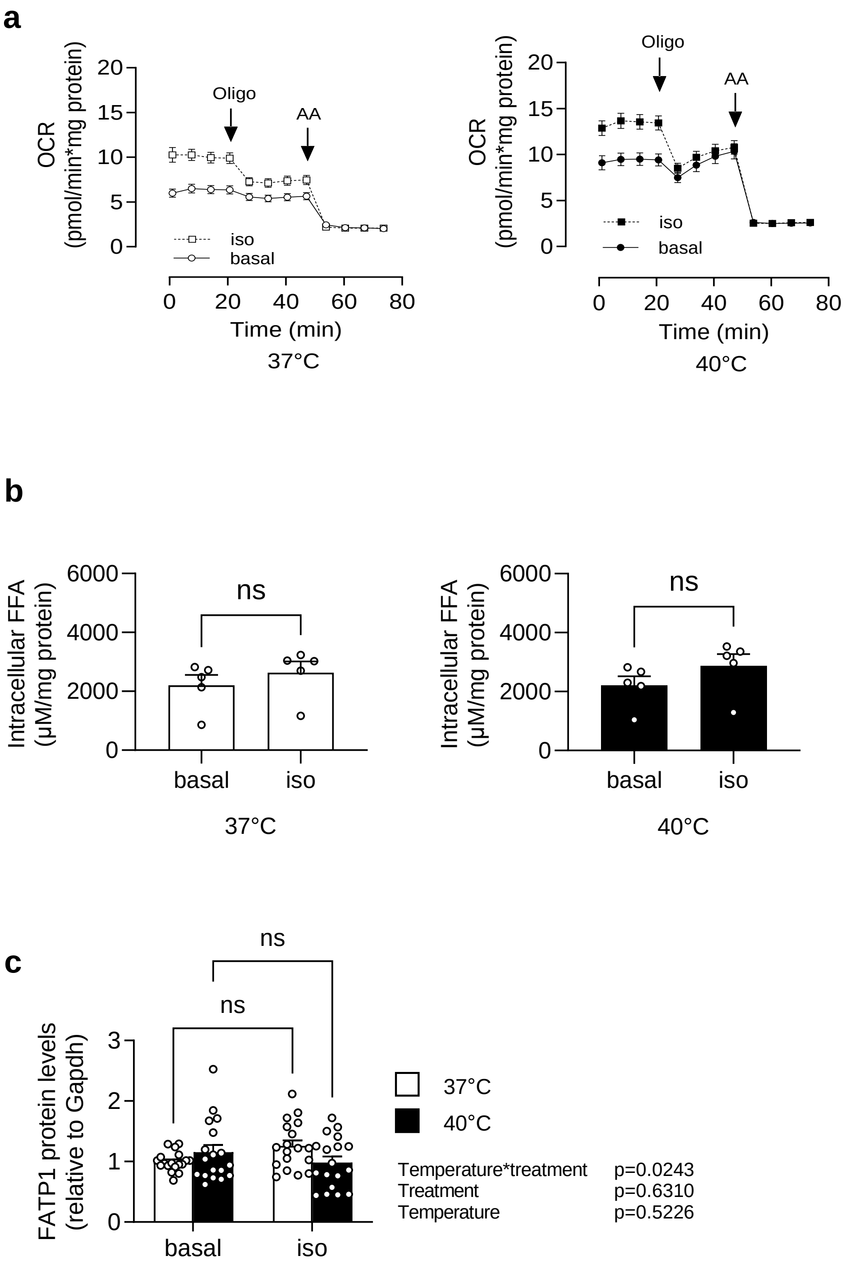

No difference in intracellular FFA concentrations between 3T3-1 adipocytes incubated at 37° and 40°C

(**a**) Oxygen consumption rate (OCR) in mature 3T3-L1 adipocytes treated with vehicle control (basal) or 1 µM isoproterenol (iso) and incubated at 37° or 40°C for 1 hour. n=39-44 wells of 2 independent cell culture experiments (17-23 well per experiment). (**b**) Intracellular FFA concentration in mature 3T3-L1 adipocytes treated with vehicle control (basal) or 1 µM isoproterenol (iso) and incubated at 37° or 40°C for 1 hour. n=5 independent cell culture experiments. (**c**) Quantification of FATP1 protein levels of 3T3-L1 adipocytes treated with vehicle control (basal) or 1 µM isoproterenol (iso) and incubated at 37° or 40°C for 1 hour. n=18 wells of 6 independent cell culture experiments (3 wells per experiment). Statistical test used: Mann-Whitney (b), two-way ANOVA with Tukey's multiple comparisons (c). Data are shown as mean±SEM.
